# Serum paraoxonase-1 as biomarker for improved diagnosis of fatty liver in dairy cows

**DOI:** 10.1186/1746-6148-9-73

**Published:** 2013-04-11

**Authors:** Ayman Samir Farid, Kazuyuki Honkawa, Eman Mohamed Fath, Nariaki Nonaka, Yoichiro Horii

**Affiliations:** 1 Department of Clinical Pathology, Faculty of Veterinary Medicine, Benha University, Moshtohor, Toukh 13736, Qalyubia, Egypt; 2 Laboratory of Parasitic Diseases, Faculty of Agriculture, University of Miyazaki, Gakuen-Kibanadai, Nishi 1-1, Miyazaki 889-2192, Japan; 3 Interdisciplinary Graduate School of Medicine and Veterinary Medicine, University of Miyazaki, Gakuen-Kibanadai, Nishi 1-1, Miyazaki 889-2192, Japan

**Keywords:** Paraoxonase, Arylesterase, Lactonase, Fatty liver, Dairy cow, Diagnosis, Liver function

## Abstract

**Background:**

Fatty liver is a major metabolic disorder in dairy cows and is believed to result in major economic losses in dairy farming due to decreased health status, reproductive performance and fertility. Currently, the definitive means for diagnosing fatty liver is determining the fat content of hepatic tissue by liver biopsy, which is an invasive and costly procedure, making it poorly suited to dairy farms. Therefore, the key aim of this study was to investigate the measurement of serum paraoxonase-1 (PON1), an enzyme exclusively synthesized by the liver, as a sensitive noninvasive biomarker for diagnosis of fatty liver in dairy cows.

**Results:**

A comparative cohort study using serum specimens from Holstein–Friesian dairy cows (46 healthy and 46 fatty liver cases) was conducted. Serum PON1 (paraoxonase, lactonase and arylesterase) activity and other biochemical and hematological parameters were measured. We found that serum PON1 activity was lower (P<0.001) in cows suffering from fatty liver. The area under the receiver operating characteristic curve (AUC-ROC) of PON1 activity for diagnosis of fatty liver was 0.973–0.989 [95% confidence interval (CI) 0.941, 1.000] which was higher than the AUC-ROC of aspartate aminotransferase (AST), lecithin-cholesterol acyltransferase (LCAT), alkaline phosphatase (ALP), non-esterified fatty acids (NEFA), beta-hydroxybutyrate (BHBA), total cholesterol, high-density lipoprotein (HDL) and low-density lipoprotein (LDL). We found that adding serum PON1 measurement to different batteries of serum diagnostic panels showed a combination of high sensitivity, specificity, positive predictive value (PPV), negative predictive value (NPV), positive likelihood ratio (+LR), negative likelihood ratio (−LR), diagnostic odd ratio (DOR) and overall diagnostic accuracy in diagnosing fatty liver.

**Conclusions:**

The present results indicate that addition of serum PON1 activity measurement to the biochemical profile could improve the diagnosis of fatty liver in dairy cows, which would have a considerable clinical impact and lead to greater profitability in the dairy industry.

## Background

Fatty liver is a major metabolic disorder of transition dairy cows affecting approximately half of the herd immediately after calving [1]. It is commonly associated with reduced productivity, fertility and immune functions, and may even lead to liver failure and premature death [2,3]. Almost all high-producing dairy cows are in negative energy balance in early lactation, as energy requirements exceed feed consumption capacity [4]. Therefore, fatty liver mainly occurs in the first 4 weeks after calving [5,6], when the hepatic uptake of lipids exceeds the oxidation and secretion of lipids by the liver. As compared with other species, the bovine liver is inefficient at exporting triglyceride (TG) in very low-density lipoprotein (VLDL) [2]. This state leads to TG accumulation in the liver, resulting in impaired hepatic metabolism [3]. Although, cows with mild and moderate fatty liver do not necessarily show clinical signs, the condition has been associated with health and production problems [7]. Despite the economic importance of this disease, it is often misidentified or overlooked due to difficulties in diagnosis [8].

Paraoxonase-1 (PON1; E.C. 3.1.8.1) is a mammalian enzyme synthesized exclusively in the liver and secreted into the blood [9]. It has been shown that PON1 is able to hydrolyze specific oxidized lipids [10], leading to a reduction in oxidative stress in serum lipoproteins, macrophages and atherosclerotic lesions [11]. Two possible substrates for bovine PON1 are commonly used. The ability of PON1 to hydrolyze paraoxon was employed as a method for measuring paraoxonase activity. PON1 also shows arylesterase activity, with phenyl acetate being one of its best substrates [12]. Moreover, in humans and several experimental studies, PON1 has shown good lactonase activity, hydrolyzing a wide range of lactones [13].

As compared with the substantial information available on PON1 from human and experimental animals’ studies, little is known about its roles in cows [14,15]. As the liver plays a key role in the synthesis of serum PON1, it is plausible that there is an association between serum PON1 activity and liver impairment during fatty liver development in dairy cows.

Standard biochemical tests for liver dysfunction are insufficiently sensitive for a reliable diagnosis of fatty liver in dairy cows; thus, histological examination of liver biopsy for determining fat contents has become the definitive means of diagnosing fatty liver [2], but this is an invasive and costly procedure, making it poorly suited for dairy farms. Therefore, finding a sensitive and noninvasive measurement for diagnosis of fatty liver is important for greater profitability in the dairy industry. The purpose of this study was to evaluate the diagnostic power of serum PON1 activity, alone and in combination with standard liver function tests, in the diagnosis of fatty liver in dairy cows.

## Methods

### Animals and serum sampling

The study population included 46 non-pregnant clinically healthy Holstein-Friesian dairy cows (aged 3–4 years) and 46 non-pregnant Holstein-Friesian dairy cows (of the same age) suffering from fatty liver. All cows were within 4 weeks postpartum. All healthy cows were confirmed by physical, serum biochemical and hematological examinations. With regard to selection of cows with fatty liver, three crucial aspects must be considered: exclusion of cases with any other pathological conditions such as fever, abomasal displacement, mastitis, metritis, vaginal discharge, and bone fractures; confirmation of fatty liver cases was performed by postmortem examination by expert slaughter house inspectors; and none of the fatty liver cases suffered from recumbency before slaughter. All cows were kept on the same farm on Kyushu Island, Japan. Blood samples were collected from each cow before feeding in the morning from the jugular vein into Vacutainer® (Terumo, Tokyo, Japan) tubes with potassium EDTA (for hematological analysis) and without anticoagulant (for serum separation). Tubes were placed in an icebox and carried to the laboratory within less than 1 h of collection. After clotting for 2 h on ice, samples were centrifuged at 1600× g for 15 min. Farm owner gave informed consent for cows to be included in the study and undergo the testing procedures. The laboratory protocols were performed in compliance with the Institutional Review Board for Animal Experiments of the University of Miyazaki.

### Hematological parameters measurement

Hematological variables including white blood cells (WBCs) and red blood cells (RBCs), hemoglobin (Hgb) concentration, hematocrit (Hct) value, mean corpuscular volume (MCV), mean corpuscular hemoglobin (MCH), and mean corpuscular hemoglobin concentration (MCHC) were determined using an electronic particle hematology analyzer (Erma PCE-210N; Diamond Diagnostic, Holliston, MA, USA).

### Biochemical parameters assay

The following parameters were assayed: serum aspartate aminotransferase (AST), gamma glutamyltranspeptidase (GGT) and alkaline phosphatase (ALP) (Dri-Chem 3500 V; Fuji-Film Co., Tokyo, Japan) [16]; non-esterified fatty acids (NEFA) (Wako Pure Chemical Ind., Osaka, Japan); β-hydroxybutyrate (BHBA) (Sanwa Kagaku Kenkyusho Co., Ltd., Nagoya, Japan); and triglyceride (TG), total cholesterol (T.CHOL), high-density lipoprotein (HDL), free cholesterol (FC) and phospholipids (PL) (Wako Pure Chemical Ind., Osaka, Japan); total protein (TP), albumin (ALB), total bilirubin (T. Bil), glucose (Glu), blood urea nitrogen (BUN), calcium (Ca), phosphorus (P), and magnesium (Mg) (Dri-Chem 7000 V; Fuji-Film Co.) [16]. Low-density lipoprotein (LDL) was calculated using the Friedewald equation [17], and VLDL was calculated using the following formula: triglyceride/5 [18]. Cholesteryl ester (CE) was calculated as T.CHOL minus FC and ester (E) ratio was calculated by dividing CE value by T.CHOL value. Albumin/globulin (AG) ratio was calculated by dividing albumin value by the globulin value.

### PON1 activities measurement

Serum PON1 activity was assayed using three synthetic substrates: paraoxon (diethyl-p-nitrophenyl phosphate; Sigma Chemical Co., St. Louis, MO, USA); and dihydrocoumarin (Sigma Chemical Co.); phenyl acetate (Nacalai Tesque, Inc, Kyoto, Japan) [19]. Briefly, PON1 activity against paraoxon was determined based on the initial rate of substrate hydrolysis to p-nitrophenol by monitoring the absorbance at 412 nm for serum samples in 100 mM Tris–HCl buffer containing 2 mM paraoxon and 2 mM CaCl2 (pH 8.0). Enzyme activity was calculated from E412 of p-nitrophenol (18,290 M−1 cm−1) and expressed in terms of U/mL (where 1 U of enzyme hydrolyses 1 nmol of paraoxon/min), while the enzyme activities toward dihydrocoumarin and phenyl acetate were determined by measuring the initial rate of substrate hydrolysis within the assay mixture containing serum samples in 100 mM Tris–HCl buffer containing 2 mM substrates, and 2 mM CaCl2 in (pH 8.0). Absorbance was monitored for dihydrocoumarin and phenyl acetate at 270 nm and enzyme activity was calculated from the E270 of dihydrocoumarin (1295 M−1 cm−1) and phenyl acetate (1310 M−1 cm−1). Enzyme activities were expressed in U/mL (where 1 U of enzyme activity hydrolyses 1 μmol of substrate/min). All activities were measured at 25°C.

### LCAT activity assay

Serum LCAT activity was assayed using the Calbiochem Fluorometric LCAT assay kit (EMD Bioscience, San Diego, CA, USA) according to the manufacturer’s instructions with some modifications. Briefly, aliquots (3 μL) of serum in both control and fatty liver cases were incubated at 37°C with the fluorescently labeled cholesterol in assay buffer containing 150 mM NaCl, 10 mM Tris–HCl, 4 mM β-mercaptoethanol and 1 mM EDTA at pH 7.4. Total assay volume was 45 μL. After 8 h of incubation at 37°C, 30 μL of the reaction mixture was added to 90 μL of the READ reagent (150 mM NaCl, 10 mM Tris–HCl, and 1 mM EDTA at pH 7.4). Conversion of cholesterol (Em. 470 nm) to cholesteryl ester (Em. 390 nm) at 340 nm excitation was determined in a fluorescence microplate reader (VICTOR Multilabel Plate Reader, PerkinElmer, Inc., Waltham, MA, USA). The change in ratio of the two intensities (470/390) was then calculated.

### Statistical analysis

We used ShapiroWilks test to examine whether variables were distributed normally. Following data distribution, differences between study groups were tested by using independent samples t-test and Mann–Whitney U test. Correlation coefficients were calculated by Spearman’s method. Partial correlation coefficients were calculated among selected blood variables, corrected for animal groups. Diagnostic accuracy for serum PON1 and other biochemical tests was calculated with receiver operating characteristic (ROC) curve analysis [20,21]. Logistic regression was used to estimate the ability of groups of variables to predict the presence or absence of liver disease. Sensitivity, specificity, positive predictive value (PPV), negative predictive value (NPV), positive likelihood ratio (+LR), and negative likelihood ratio (−LR) were computed with the corresponding 95% confidence intervals (CI). We also calculated the diagnostic odds ratio (DOR) which expresses the strength of the association between the test result and disease [22]. Statistical analysis was performed using the statistical software package SPSS for Windows (version 18.0; SPSS Inc., Chicago, IL, USA). Statistical significance between mean values was set at P < 0.05. Data are reported as means and standard deviations (SD).

## Results

### Clinical signs

No clinical signs were observed in the control group at the time of sample collection. On the other hand, the diseased cases showed decreased appetite, rough fur, weak ruminal movement, loss of abdominal circumference, decreased milk yield, rapid deterioration of body conditions and sometimes watery diarrhea.

### Biochemical and hematological parameters

Table 1 shows the biochemical and hematological parameters in healthy cows and cases with fatty liver. Serum PON1 (paraoxonase, lactonase and arylesterase) activity was significantly reduced (−53%, –56%, and −36%, respectively; P<0.001), in cases with fatty liver, as compared to controls.

**Table 1:** Serum biochemical and hematological parameters from healthy reference cows and cows with fatty liver

	**Control ( *n *=46)**	**Fatty liver ( *n *=46)**	**% change**	**Distribution**	***P *value ≤**
**Biochemical parameters**					
Paraoxonase (U/mL)	**82.11 ± 22.42**	**38.47 ± 13.40**	**−53.14**	**0.029**	**0.001**
Lactonase (U/mL)	**5.82 ± 1.70**	**2.56 ± 1.11**	**−55.86**	**0.043**	**0.001**
Arylesterase (U/mL)	**47.92 ± 7.36**	**30.44 ± 7.88**	**−36.48**	**0.028**	**0.001**
AST (U/L)	**67.84 ± 14.53**	**136.78 ± 68.14**	**101.62**	**0.000**	**0.001**
GGT (U/L)	**44.18 ± 10.43**	**49.93 ± 30.25**	**13.02**	**0.068**	**0.903**
ALP (U/L)	**232.45 ± 73.10**	**225.69 ± 84.07**	**−2.907**	**0.048**	**0.871**
LCAT (470/390)	**3.80 ± 1.10**	**3.20 ± 0.84**	**−15.81**	**0.000**	**0.015**
T. Bil (μmol/L)	**5.30 ± 2.22**	**18.31 ± 7.69**	**245.28**	**0.000**	**0.001**
NEFA (mmol/L)	**0.22 ± 0.20**	**1.19 ± 0.60**	**440.90**	**0.000**	**0.001**
BHBA (mmol/L)	**0.725 ± 0.31**	**2.94 ± 1.77**	**305.51**	**0.000**	**0.001**
T.CHOL (mmol/L)	**4.71 ± 1.17**	**1.58 ± 0.61**	**−66.45**	**0.171**	**0.001**
HDL (mmol/L)	**1.94 ± 0.71**	**1.19 ± 0.001**	**−38.65**	**0.048**	**0.001**
VLDL (mmol/L)	**0.141 ± 0.05**	**0.08 ± 0.05**	**−43.26**	**0.808**	**0.001**
LDL (mmol/L)	**2.72 ± 1.71**	**0.44 ± 0.37**	**−83.82**	**0.001**	**0.001**
TG (mmol/L)	**0.30 ± 0.12**	**0.39 ± 0.14**	**30.00**	**0.808**	**0.002**
FC (mmol/L)	**0.94 ± 0.35**	**0.37 ± 0.15**	**−60.63**	**0.135**	**0.001**
CE (mmol/L)	**3.84 ± 1.49**	**1.20 ± 0.53**	**−68.75**	**0.177**	**0.001**
E. ratio	**80.12 ± 3.73**	**75.12 ± 9.95**	**−6.24**	**0.000**	**0.014**
PL (mmol/L)	**2.89 ± 1.05**	**0.85 ± 0.29**	**−70.58**	**0.036**	**0.001**
TP (g/L)	**70.92 ± 5.60**	**69.10 ± 6.8**	**−2.56**	**0.005**	**0.749**
ALB (g/L)	**35.5 6 ± 2.56**	**32.59 ± 3.31**	**−8.565**	**0.018**	**0.001**
AG ratio	**1.032 ± 0.19**	**0.91 ± 0.18**	**−11.614**	**0.570**	**0.004**
GLU (mmol/L)	**2.55 ± 0.61**	**2.74 ± 0.15**	**7.45**	**0.004**	**0.739**
BUN (mmol/L)	**12.69 ± 8.49**	**5.40 ± 6.70**	**−57.44**	**0.000**	**0.083**
Ca (mmol/L)	**2.56 ± 0.15**	**2.21 ± 0.27**	**−13.67**	**0.000**	**0.001**
P (mmol/L)	**1.92 ± 0.31**	**2.05 ± 0.90**	**6.77**	**0.462**	**0.657**
Mg (mmol/L)	**1.05 ± 0.12**	**0.90 ± 0.12**	**−14.28**	**0.000**	**0.001**
**Hematological parameters**					
WBCs (10^9^/L)	**8.045 ± 1.88**	**8.87 ± 5.48**	**10.267**	**0.000**	**0.188**
RBCs (10^12^/L)	**5.94 ± 0.67**	**5.35 ± 1.18**	**−9.951**	**0.937**	**0.002**
Hgb (g/L)	**98.52 ± 10.13**	**98.17 ± 21.60**	**−0.441**	**0.637**	**0.795**
HCT%	**26.86 ± 4.26**	**26.90 ± 2.16**	**0.138**	**0.015**	**0.924**
MCV (fL)	**45.65 ± 4.95**	**50.21 ± 6.14**	**9.990**	**0.239**	**0.001**
MCH (pg)	**16.65 ± 1.70**	**18.33 ± 1.57**	**10.132**	**0.948**	**0.001**
MCHC (g/L)	**36.66 ± 2.84**	**35.96 ± 5.66**	**−1.909**	**0.284**	**0.478**
PLT (10^9^/L)	**398.28 ± 114.99**	**351.36 ± 99.68**	**−11.779**	**0.484**	**0.001**

Data are presented as mean values ± standard deviation of mean.

AST, aspartate aminotransferase; GGT, gamma glutamyltransferase; ALP, alkaline phosphatase; LCAT, lecithin-cholesterol acyltransferase; T. Bil, total bilirubin; NEFA, non-esterified fatty acids; BHBA, beta-hydroxybutyrate; T.CHOL, total cholesterol; HDL, high-density lipoprotein; VLDL, very low-density lipoprotein; LDL, low-density lipoprotein; TG, triglyceride; FC, free cholesterol; CE, cholesteryl ester; E. ratio, ester ratio; PL, phospholipids; TP, total protein; ALB, albumin; AG, albumin/globulin ratio; GLU, Glucose; BUN, blood urea nitrogen; Ca, total serum calcium; P, inorganic phosphorus; Mg, magnesium; WBC, total white blood cell count; RBC, total red blood cell count; Hg, hemoglobin content; HCT, hematocrit; MCV, mean corpuscular volume; MCH, mean corpuscular hemoglobin; MCHC, mean corpuscular hemoglobin concentration; and PLT, platelet count.

Other biochemical parameters supported the presence of fatty liver, as manifested by significant increases in AST (101%; P<0.001), NEFA (440%; P<0.001), BHBA (305%; P<0.001), total bilirubin (T. Bil) (245%; P<0.001) and TG (30%; P<0.01). Fatty liver cases also showed significant decreases in LCAT (−16%; P<0.05), T.CHOL (−66%; P<0.001), HDL (−38%; P<0.001), VLDL (−43%; P<0.001), LDL (−83%; P<0.001), FC (−60%; P<0.001), CE (−68%; P<0.001), PL (−70%; P<0.001), E. ratio (−6%; P<0.05), ALB (−8%; P<0.001) and A/G ratio (−11%; P<0.01). In addition, significant hypocalcemia (P<0.001) and hypomagnesmia (P<0.001) were associated with fatty liver cases. There were non-significant changes in the concentrations of total protein, glucose, blood urea nitrogen and phosphorus.

For hematology parameters, cases with fatty liver showed a significant (P<0.01) decrease in the number of RBCs, as well as significant (P<0.001) increases in the volume of RBCs and hemoglobin content, as indicated by increased MCV and MCH, leading to a state of macrocytic anemia.

**Figure 1:**
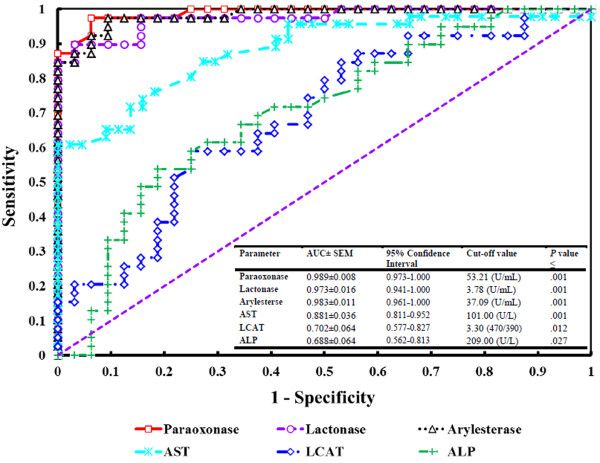
**Receiver operating characteristic (ROC) curves for diagnostic efficacies of PON1 and some other liver enzymes.** ROC curves were evaluated for serum PON1 (paraoxonase, arylesterase and lactonase), AST, ALP and LCAT activities in the prediction of fatty liver in dairy cow. AUC, Area under ROC curve; SEM, standard error of mean.

**Figure 2:**
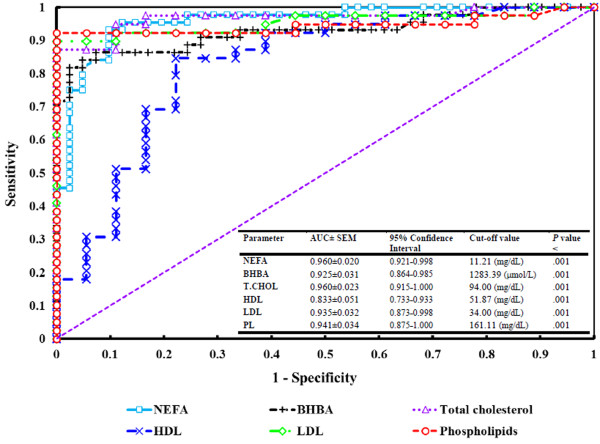
**Receiver operating characteristic (ROC) curves for diagnostic efficacies of serum lipid metabolism indicators.** ROC curves were evaluated for serum NEFA, BHBA, total cholesterol, HDL, LDL and phospholipids in the prediction of fatty liver in dairy cow. AUC, Area under ROC curve; SEM, standard error of mean.

Diagnostic performance was evaluated by ROC analysis with pertinent areas under the curve (AUCs). AUCs with the corresponding cut-off and p values are listed in Figures 1 and 2. Curves for paraoxonase, lactonase, arylesterase, AST, ALP and LCAT appear in Figure 1. The AUCs for PON1 (paraoxonase, lactonase and arylesterase) activity were 0.989, 0.983 and 0.973 respectively, which were higher than those of other liver enzymes (AST, LCAT and ALP showed AUCs of 0.881, 0.702 and 0.688, respectively). NEFA, BHBA, total cholesterol, HDL, LDL and phospholipids showed AUCs of 0.960, 0.925, 0.960, 0.833, 0.935 and 0.941, respectively (Figure 2).

In order to further illustrate the correlation coefficients of the regression lines between serum PON1 activity and the other biochemical analytes, Spearman and partial correlation coefficients are depicted in Table 2 below and above the diagonal, respectively. Spearman correlation (below the diagonal) showed significant correlations between paraoxonase activity and indicators of liver damage or dysfunction [AST (r=−0.50; P<0.01), total bilirubin (r=−0.65; P<0.01) and LCAT (r=0.27; P<0.05)], with indicators of fat mobilization [NEFA (r=−.66; P<0.01), and BHBA (r=−0.56; P<0.01)], as well as significant correlations with indicators of hepatic synthesis and metabolic abilities [albumin (r=0.49; P<0.01), total cholesterol (r=0.82; P<0.01), HDL (r=0.62; P<0.01), VLDL (r=0.32; P<0.01), LDL (r=0.72; P<0.01), triglycerides (r=0.24; P<0.05), free cholesterol (r=0.79; P<0.01), and cholesterol ester (r=0.81; P<0.01)] support the notion that PON1 activity is a good reflection of hepatic function. Notably, the three activities of PON1 (paraoxonase, lactonase and arylesterase) in dairy cow showed significant positive correlations (r=0.96–97; P<0.01) with one another. On the other hand, when the animal health conditions were controlled, partial correlation coefficients (above the diagonal), showed that there are no correlations between paraoxonase and the indicators of liver damage and fat mobilization (AST, LCAT, total bilirubin, NEFA and BHBA). We can infer that the relationship between PON1 activity and liver damage or dysfunction biomarkers is mediated by fatty liver.

**Table 2:** Spearman and partial correlation coefficients for selected blood variables from healthy cows and cows with fatty liver below and above the diagonal, respectively

	**Par**	**Lact**	**Aryl**	**AST**	**ALP**	**LCAT**	**TBil**	**NEFA**	**BHBA**	**T.CHOL**	**HDL**	**VLDL**	**LDL**	**TG**	**FC**	**CE**	**PL**	**TP**	**ALB**	**BUN**	**Ca**	**Mg**
**Para**	**1**	.96**	.83**							.49**	.37**		.36**		.45**	.48**	.40**		.23*			
**Lact**	.97**	**1**	.85**						.22*	.49**	.32**		.38**		.42**	.48**	.38**		.27*			
**Aryl**	.97**	.96**	**1**				.23*	.29*	.31*	.44**	.43**		.28*		.38**	.44**	.32**		.33**			
**AST**	-.50**	-.48**	-.47**	**1**	.25*																	
**ALP**	.33**	.31**	.34**		**1**						.32**											
**LCAT**	.27*	.28**	.28**			**1**				.51**			.61**		.41**	.51**	.43**					
**TBil**	-.65**	-.64**	-.63**	.56**	-.31**		**1**	.63**	.50**													
**NEFA**	-.66**	-.65**	-.65**	.64**	-.35**	-.23*	.83**	**1**	.52**													
**BHBA**	-.56**	-.55**	-.55**	.67**			.76**	.72**	**1**										.37**			
**T.CHOL**	.82**	.81**	.81**	-.55**	.34**	.37**	-.70**	-.70**	-.58**	**1**	.39**		.90**		.87**	.99**	.80**		.46**			
**HDL**	.62**	.60**	.63**	-.38**	.52**		-.50**	-.54**	-.45**	.74**	**1**				.31**	.40**	.35**		.41**		.31**	.27*
**VLDL**	.32**	.30*	.30**	-.37**			-.36**	-.25*	-.33**	.44**		**1**										
**LDL**	.72**	.73**	.70**	-.50**		.50**	-.57**	-.62**	-.44**	.89**	.43**	.44**	**1**		.80**	.89**	.71**		.30*			
**TG**	.24*	.22*	.25*	-.26*			-.29**			.33**		1**	.44**	**1**		.81**	.72**		.29*			
**FC**	.79**	.76**	.76**	-.54**	.32**	.35**	-.64**	-.66**	-.53**	.88**	.63**	.42**	.84**	.29**	**1**							
**CE**	.81**	.80**	.81**	-.55**	.35**	.35**	-.69**	-.70**	-.58**	.99**	.74**	.43**	.89**	.32**	.83**	**1**	.79**		.48**			
**PL**	.77**	.76**	.76**	-.48**	.28*	.38**	-.69**	-.67**	-.55**	.91**	.64**	.37**	.83**	.25*	.84**	.89**	**1**		.36**	-.33**		
**TP**																		**1**	.24*			
**ALB**	.49**	.50**	.52**				-.30**	-.27*		.55**	.56**	.30**	.55**	.24*	.49**	.56**	.50**	.38**	**1**		.24*	.39**
**BUN**			.23*		.31**									.26*		.22*			.37**	**1**		
**Ca**	.67**	.65**	.66**	-.47**	.43**		-.62**	-.53**	-.53**	.68**	.67**	.39**	.60**	.29**	.63**	.69**	.64**		.59**		**1**	
**Mg**	.46**	.46**	.46**	-.43**	.35**		-.59**	-.55**	-.56**	.58**	.47**	.31**	.41**	.21*	.48**	.59**	.51**		.41**	.25*	.55**	**1**

All correlations shown are significant (*P<0.05, **P<0.01).

The usefulness of adding serum PON1 measurement to the biochemical panel of liver function tests was analyzed by multiple logistic regression analysis. This showed that adding PON1 values to other biochemical results increases the sensitivity, specificity, PPV, NPV and likelihood ratios (positive and negative), as well as overall diagnostic accuracy. The optimum diagnostic cut off point maximizing the sensitivity and specificity was determined to be 53.21 U/mL for paraoxonase activity, with a sensitivity of 86.7% and specificity of 93.5%. The corresponding PPV and NPV levels were 93.48% and 86.67%, respectively. Adding PON measurement to other biochemical parameters improves the diagnostic indices by increasing +LR, decreasing -LDR, and greatly increasing DOR. As shown in Table 3, the best performance was observed with a combination of paraoxonase and NEFA measurements, and paraoxonase, NEFA, BHBA, AST, LCAT and total cholesterol.

**Table 3:** Performance characteristics of best variables for diagnosis of fatty liver

**Test/panel of tests**	**Sensitivity**		**Specificity**		**PPV**		**NPV**		**+LR**		**–LR**		**DOR**		**Accuracy**
	**%**	**95% CI**	**%**	**95% CI**	**%**	**95% CI**	**%**	**95% CI**		**95% CI**		**95% CI**		**95% CI**	**%**
**PON**	**86.7**	**75.7–94.2**	**93.5**	**80.9 –97.5**	**92.8**	**81.0–98.3**	**87.7**	**72.5–94.4**	**12.2**	**4.1–36.7**	**0.1**	**0.0–0.2**	**93.1**	**21.8 –398.0**	**90.1**
**NEFA**	**92.8**	**81.3 –97.6**	**86.9**	**72.5–98.1**	**86.6**	**73.0–94.5**	**92.0**	**79.4–98.1**	**6.9**	**3.2–14.7**	**0.0**	**0.0–0.2**	**86.6**	**20.2–371.0**	**89.7**
**NEFA+PON**	**99.7**	**89.6–99.8**	**95.2**	**83.0–99.1**	**95.4**	**83.7–99.1**	**99.7**	**89.2–99.8**	**21.0**	**5.6–78.6**	**0.0**	**0.0 –1.1**	**8440.0**	**14.6–4871511.6**	**97.7**
**NEFA+BHBA**	**92.8**	**81.3–97.6**	**90.9**	**77.4–96.1**	**90.6**	**77.4–97.0**	**93.0**	**79.4–98.1**	**9.5**	**3.7–24.2**	**0.0**	**0.0 – 0.2**	**123.3**	**25.8–588.2**	**91.8**
**NEFA+BHBA+PON**	**97.6**	**87.9–99.5**	**95.4**	**83.8–98.6**	**95.3**	**83.3–99.2**	**97.6**	**85.9–99.8**	**20.0**	**5.1–77.4**	**0.0**	**0.0–0.1**	**819.0**	**71.4–9393.6**	**96.5**
**NEFA+BHBA+AST**	**92.5**	**79.0–98.1**	**90.2**	**75.9–96.8**	**90.2**	**77.4–97.0**	**93.0**	**78.5–98.0**	**9.7**	**3.8–24.7**	**0.0**	**0.0–0.2**	**166.5**	**28.6–966.1**	**91.6**
**NEFA+BHBA+AST+PON**	**97.5**	**86.8–99.5**	**95.4**	**83.5–98.6**	**95.1**	**83.3–99.2**	**97.6**	**85.2–99.8**	**19.4**	**5.0–75.3**	**0.0**	**0.0–0.1**	**722.0**	**62.7–8301.8**	**96.4**
**NEFA+BHBA+AST+LCAT**	**94.8**	**82.7–98.5**	**90.0**	**77.4–96.1**	**90.2**	**75.4–96.7**	**94.7**	**81.3–99.1**	**9.7**	**3.8–24.7**	**0.0**	**0.0–0.2**	**166.5**	**28.6–966.1**	**92.4**
**NEFA+BHBA+AST+LCAT+PON**	**97.4**	**86.8-99.5**	**95.0**	**83.5–98.6**	**95.0**	**81.7–99.1**	**97.4**	**84.9–99.8**	**19.4**	**5.0–75.3**	**0.0**	**0.0–0.1**	**722.0**	**62.7–8301.8**	**96.2**
**NEFA+BHBA+AST+LCAT+T.CHOL**	**97.4**	**86.8–99.5**	**95.0**	**83.5–98.6**	**95.0**	**81.7–99.1**	**97.4**	**84.9–99.8**	**19.4**	**5.0–75.3**	**0.0**	**0.0–0.1**	**722.0**	**62.7–8301.8**	**96.2**
**NEFA+BHBA+AST+LCAT+T.CHOL+PON**	**99.7**	**88.4–99.8**	**97.2**	**84.9–99.8**	**97.2**	**84.9–99.8**	**99.7**	**88.4–99.8**	**36.4**	**–5.7–230.2**	**0.0**	**0.0–1.2**	**13898.2**	**21.1–9140510.2**	**98.7**

NPV, negative predictive value; PPV, positive predictive value; +LR, positive likelihood ratio; –LR, negative likelihood ratio; DOR, diagnostic odd ratio.

The cut-off values used for the tests were ≤ 53.22, 0.39, 1.28, 101.00, 3.31, 2.43 for PON (U/mL), NEFA (mmol/L), BHBA (mmol/L), AST (U/L), LCAT (470/390) and T.CHOL (mmol/L), respectively.

## Discussion

The liver plays a pivotal role during the transition period of dairy cows, and its functions are necessary for successful adaptation without the occurrence of health disorders [23]. Therefore, finding suitable noninvasive tests for improving the evaluation of liver function and aiding in diagnosis of fatty liver is urgently necessary.

There are no standardized methods for measuring PON1 esterase activity. The most widely used method is the hydrolysis of paraoxon. However, this method is not free of drawbacks. Firstly, paraoxon is very unstable and extremely toxic, which is not suitable for a high-throughput routine method. Secondly, the interpretation of the physiological significance of a measurement conducted with such an unnatural substrates is questionable. In addition, the enzyme’s activity on paraoxon is strongly influenced by the PON1_192_ genetic polymorphism (which is not well studied in bovine) [24]. Recent significant advances in the search for reliable PON1 lactonase activity assays may facilitate the measurement in a routine clinical chemistry laboratory setting [25]. This study is the first to measure lactonase activity in bovine samples, which makes PON1 measurement closer to practical development in veterinary field. In the present study, we found that serum PON1 (paraoxonases, lactonase and arylesterase) activity is significantly reduced in cows with fatty liver, as compared with controls. Several mechanisms may explain this decrease in serum PON1 activity. First, PON1 activity may be decreased as a result of altered PON1 synthesis by the liver [26,27], possibly due to increased hepatic and serum proinflammatory cytokine (TNF-α) in cows with fatty liver [2,28]. Second, reductions in serum PON1 activity in fatty liver cases might be attributed to changes in HDL structure and composition [29], and this is supported by our findings of decreased LCAT activity in fatty liver cases, which is in accordance with the results of Nakagawa et al., (1997) [30]. Both PON1 and LCAT are HDL-associated proteins. Accordingly, the reduction in serum PON1 and LCAT activities appears to be mediated by alterations in HDL structure and concentration, which may be primarily caused by fatty liver [31]. Finally, cows with fatty liver showed increased free radical production manifested by high levels of malondialdehyde [32], and PON1 is known to be inactivated after hydrolysis of lipid peroxides [33].

In normal dairy cows, lipid accumulates in the liver chiefly in the form of triglycerides, this accumulation reaches up to 10 times the normal value in cows suffering from fatty liver [8,34]. It is conceivable that this accumulation of triglycerides in the cytoplasm of hepatocytes causes disturbances in hepatic structure and function [34]. Enzyme leakage from hepatocytes is one manifestation of these disturbances. Serum AST increases significantly in accordance with serious hepatic damage induced by fatty accumulation [28]. This is supported by significant positive correlations between AST and NEFA (r=0.64; P<0.01) and BHBA (r=0.67; P<0.01). Although AST activity is relatively high and in similar amounts in liver and in skeletal and cardiac muscle, suggesting that it is not liver specific [35], but its diagnostic sensitivity is reported to be 94% for fatty liver in dairy cows with a strong correlation between serum activity and liver triglyceride content [36]. On the other hand, GGT is almost entirely associated with the biliary epithelium in the liver and is consequently not released into circulation until bile damage occurs. It seems likely that fat infiltration, during fatty liver development in our study, does not induce either cholestasis or bile duct damage, as no significant correlations were observed between GGT and other biochemical parameters. This is supported by non-significant changes in serum ALP concentration instead of the expected increases. Taken together, the results for GGT and ALP values indicate that fat infiltration in the liver does not induce intraluminal pressure leading to intrahepatic obstruction of the bile ducts. We also found significant hyperbilirubinemia in the fatty liver group, which suggests liver damage caused by increased unconjugated bilirubin [37]. The higher bilirubin levels observed in fatty liver group could be also explained with a lower liver synthesis of the enzymes necessary to remove bilirubin from the blood; these enzymes could be also considered as negative acute phase proteins [38].

We found a significant increase in the concentration of serum NEFA levels. Elevated NEFA concentrations are a common factor in the induction of fatty liver, reflecting excessive mobilization of body fat to cope with the high energy demand of milk synthesis. Under such conditions, which are associated with marked formation of acetyl coenzyme A, the tricarboxylic acid cycle cannot fully metabolize fatty acids. As a result, acetyl coenzyme A is converted to acetoacetate, which is then reduced to BHBA by BHB dehydrogenase or spontaneously decarboxylized to acetone [39]. This explains our observation of a significant increase in the concentration of BHBA in fatty liver cases. This increase in BHBA concentration is known to precede and be associated with the development of fatty liver [40].

As the liver occupies a central position in bovine metabolism, fatty liver is accompanied by disturbances in hepatic structure and function [34]; therefore, cows with fatty liver may also show abnormal lipid and lipoprotein concentrations [41]. In our study, cows with fatty liver showed a significant decrease in total cholesterol, which was inversely related to NEFA concentrations. These results are in accordance with previous reports [8]. The decrease in serum HDL may have resulted from impaired hepatic secretion of apolipoprotein A, the basic protein for the synthesis of HDL, that is considered a negative acute phase protein, and thus the reduction in HDL could be caused by an inflammatory condition [3,42] and may be related to the lower cholesterol levels seen in cows with fatty liver, as HDL consists of about 60% cholesterol [43].

We also found a significant decrease in VLDL in the fatty liver group, as compared with healthy cows. Several previous studies have noted that the accumulation of fat in the liver cells and development of fatty liver is caused by reduced synthesis of VLDL [44,45]. Decreased VLDL secretion and decreased conversion to LDL explains our findings of decreased serum LDL levels in cows with fatty liver. Another possible reason for the decreased LDL levels is the increased rate of LDL catabolism [46]. The decreased concentrations of phospholipids (fatty acyl donor in LCAT reaction) and free cholesterol (fatty acyl acceptor) observed in cows with fatty liver may be a consequence of reduced LCAT activity [3]. The decrease in CE concentration also appeared to be attributable, at least in part, to the decreased LCAT activity and other enzymes responsible for cholesterol synthesis, such as hydroxymethylglutaryl (HMGT)-CoA reductase [47]. An unexpected clinicopathological observation in our study was the significant increase, instead of the expected decrease, in plasma triglyceride concentrations in cows with fatty liver when compared with the control group. Although this observation is supported by the work of Basoglu et al., (2002) [48], it requires further investigation.

We found a significant hypoalbuminemia in fatty liver cases as a result of decreased volume of rough endoplasmic reticulum and mitochondrial damage [40] induced by fat infiltration of hepatic tissue. In addition, albumin is a negative acute phase protein and extensive inflammation may compound this hypoalbuminemia [37]. Moreover, low A/G ratio in the presence of normal total protein indicates elevated globulins in response to acute inflammation [37].

Serum glucose concentration was not significantly different between healthy and fatty liver cases. This finding may be due to insulin resistance, which commonly accompanies fatty liver in dairy cows [2]. This finding is in accordance with the results of Kalaitzakis et al. (2006) [36].

Despite the importance of histopathological examination of hepatic tissue, Japanese regulations prevented us from doing this in the present study. However, in our study we have confirmed the presence of fatty liver by postmortem examination which considered, regardless the degree of fatty liver, as a strong evidence for its presence in diseased animals. Postmortem examination of cows with fatty liver revealed swelling of the hepatic parenchyma, which increased the size and the volume of the organ [49]. Additionally, from the practical point of view, lipid profile disturbances and LCAT activity are useful serum biomarkers for diagnosis of fatty liver in cows [3] that enable us to confirm the diagnosis of fatty liver cases in our study. However, the lack of postmortem examinations on the control cows is a limitation of the study. Acknowledging such limitation of our study, future investigations are required to assess the correlation between serum PON1 activity and hepatic triglycerides in both healthy and diseased cows.

Severe fatty liver does not always result in measurable hepatic dysfunction. Due to the apparent variability in the usefulness of biopsy in determining the severity and/or diagnosis of clinical disease [6], a “battery” of tests needs to be performed to increase the sensitivity and specificity of diagnosis. Among the direct biomarkers of fatty liver, we found good diagnostic performance for NEFA, BHBA, AST and lipid profile. These biomarkers showed excellent predictive value for diagnosis of fatty liver. Notably, addition of serum PON1 to these biochemical tests gave a combination of high sensitivity, specificity, PPV, NPV, +LR, –LR, DOR and overall diagnostic accuracy in diagnosing fatty liver (Table 3). The AUC-ROC of PON1 activity for the detection of fatty liver was more than 98% accurate, which has not been achieved through other diagnostic algorithms.

Importantly, from an economic point of view, the combined measurement of PON1 activity and NEFA concentration would be useful for diagnosing cows with fatty liver as it provides sensitivity, specificity, PPV, NPV, +LR, -LR, DOR and overall diagnostic accuracy comparable to that produced by the combination of NEFA, BHBA, AST, LCAT, total cholesterol, and PON1. Moreover, the strong correlations between serum PON1 and AST, LCAT, total bilirubin, NEFA, BHBA and lipid profile make serum PON1 measurement advantageous in diagnosis of fatty liver (Table 3).

It is noteworthy that the measurement of serum PON1 activity is a simple, reliable, fast, inexpensive, readily automated method that is compatible with random access analysis in barcoded primary tubes, and can be performed with most automated analyzers used in standard biochemical liver function tests [50]. The only drawback, the use of the toxic substrate paraoxon, can be overcome using the nontoxic lactonase activity, as our results (Table 2) showed a good correlation between paraoxonase and lactonase activity in bovine serum.

## Conclusions

In conclusion, this study demonstrates that addition of serum PON1 activity measurement to the biochemical profile could improve the diagnosis of fatty liver in dairy cows. Future studies in this area should focus on the correlation between serum PON1 activity and hepatic triglycerides content in both healthy and ill dairy cows as well as the diagnostic validation of serum PON1 assay in the early prediction of fatty liver development in dairy farms, which would have a considerable clinical impact and lead to greater profitability in the dairy industry.

## Abbreviations

AG: Albumin/globulin; ALB: Albumin; ALP: Alkaline phosphatase; AST: Aspartate aminotransferase; BHBA: β-hydroxybutyrate; BUN: Blood urea nitrogen; Ca: Calcium; CE: Cholesteryl ester; EDTA: Ethylenediaminetetraacetic acid; FC: Free cholesterol; GGT: Gamma glutamyltranspeptidase; Glu: Glucose; h: Hour; Hct: Hematocrit; HDL: High-density lipoprotein; Hgb: Hemoglobin; LCAT: Lecithin-cholesterol acyltransferase; LDL: Low-density lipoprotein; MCH: Mean corpuscular hemoglobin; MCHC: Mean corpuscular hemoglobin concentration; MCV: Mean corpuscular volume; Mg: Magnesium; NEFA: Non-esterified fatty acids; P: Phosphorus; PL: Phospholipids; PON1: Paraoxonase-1; RBCs: Red blood cells; T. Bil: Total bilirubin; T.CHOL: Total cholesterol; TG: Triglyceride; TP: Total protein; VLDL: Very low-density lipoprotein; WBCs: White blood cells.

## Competing interests

The authors declare that they have no competing interests.

## Authors’ contributions

ASF and YH conceived and designed the experiments; ASF, KH, and EM performed the experiments; ASF analyzed the data; NN shared in discussing the results; and ASF and YH wrote the paper. All authors read and approved the final manuscript.
